# scFates: a scalable python package for advanced pseudotime and bifurcation analysis from single-cell data

**DOI:** 10.1093/bioinformatics/btac746

**Published:** 2022-11-17

**Authors:** Louis Faure, Ruslan Soldatov, Peter V Kharchenko, Igor Adameyko

**Affiliations:** Department of Neuroimmunology, Center for Brain Research, Medical University Vienna, 1090 Vienna, Austria; Department of Biomedical Informatics, Harvard Medical School, Boston, MA 02115, USA; Department of Biomedical Informatics, Harvard Medical School, Boston, MA 02115, USA; Altos Labs, San Diego, CA 92121, USA; Department of Neuroimmunology, Center for Brain Research, Medical University Vienna, 1090 Vienna, Austria

## Abstract

**Summary:**

scFates provides an extensive toolset for the analysis of dynamic trajectories comprising tree learning, feature association testing, branch differential expression and with a focus on cell biasing and fate splits at the level of bifurcations. It is meant to be fully integrated into the scanpy ecosystem for seamless analysis of trajectories from single-cell data of various modalities (e.g. RNA and ATAC).

**Availability and implementation:**

scFates is released as open-source software under the BSD 3-Clause ‘New’ License and is available from the Python Package Index at https://pypi.org/project/scFates/. The source code is available on GitHub at https://github.com/LouisFaure/scFates/. Code reproduction and tutorials on published datasets are available on GitHub at https://github.com/LouisFaure/scFates_notebooks.

**Supplementary information:**

Supplementary data are available at *Bioinformatics* online.

Pseudotime analysis is a concept first introduced in the early days of single-cell analysis for microarray data (Magwene *et al.*, 2003). Derived from the idea that the temporal structure of gene expression can be retrieved by looking at its geometry (Rifkin and Kim, 2002), pseudotime analysis consists in ordering cells in a single-dimensional value meant to recapitulate the underlying transcriptional transition. The advent of more sensitive scRNAseq methods was accompanied by the development of two main classes of high-resolution trajectory analysis tools. One relies on fate probability mapping of the cells, with tools such as Palantir (Setty *et al.*, 2019), and CellRank (Lange *et al.*, 2022). Such approaches have the advantages of being flexible and efficient in finding terminal states, with the possibility for manual selection of these. However, the results do not lead to an easily interpretable tree as each cell is given a probability value for each fate, without clearly defined branches or segments. The other class of tools relies on principal graph learning, with the most used tool being Monocle3 (Cao *et al.*, 2019). Principal graph derives from the concept of principal manifold, which is defined as a group of surfaces or lines that goes across the ‘middle’ of the data (Hastie, 1984). Principal graph represents a skeleton of the data that aims to capture major geometric structures, usually involving nodes connected by edges, whose both positions are optimized within the reduced dimensions to approximate the underlying expression manifold based on the positions of cells within the same expression dimensions. Reconstructing a principal graph enables generating discrete segments and bifurcations, allowing easier interpretation. However, Monocle3 learns the principal graph on Uniform Manifold Approximation and Projection (UMAP) embedding, a low-dimensional representation highly sensitive to parameters and which can represent a highly distorted view of the data (Zhai, 2022). Among general pseudotime toolsets, both Monocle3 and STREAM (Chen *et al.*, 2019) have functions for gene-pseudotime association testing, with STREAM going even further by providing branch differential expression (DE) related functions. However, no tool performs statistical analysis of the bifurcation point, including the discovery of early cell fate biasing factors (Bargaje *et al.*, 2017).

Here, we introduce scFates, a python package to streamline the whole process of pseudotime analysis, with flexible tree learning options, advanced feature extraction tasks and specific functions focused on bifurcation analysis. scFates was initially based on crestree R package, developed for the analysis of cell fate dynamics in development (Faure *et al.*, 2020; Krivanek *et al.*, 2020; Soldatov *et al.*, 2019). While the initial R version included a tree inference approach inspired by SimplePPT (Mao *et al.*, 2015). scFates additionally implements ElPiGraph, another method for principal graph learning (Albergante *et al.*, 2020), allowing investigators to impose topological constraints on trajectories to fit single curves or circular trajectories. scFates is fully compatible with the scanpy ecosystem (Wolf *et al.*, 2018) by using the anndata format and provides Graphics Processing Unit (GPU) and multicore accelerated functions for faster and more scalable inference. scFates is divided into three main parts: (i) trajectory learning and graph operations, (ii) feature (transcript counts, ATAC peaks,…) significance over pseudotime testing and clustering and (iii) bifurcation analysis. All three parts implement publication-grade plotting capabilities to visualize the results.

The trajectory learning can be performed on processed single-cell data at different stages of analysis, such as on normalized transcript count matrix, principal components or diffusion maps. The user has the choice of running either ElPiGraph or SimplePPT algorithms to learn a principal graph. ElPiGraph generates more robust structures by taking advantage of graph grammars and multiple topologies exploration at each iteration, at the cost of a lower scaling with the number of nodes (up to 50). When the transition is known to be non-bifurcating, simpler constrained curves can be learned instead. For example, a principal circle can be inferred for cell cycle analysis. While ElPiGraph can be used for higher number of nodes (up to 1000) by checking only one topology per iteration, we found that SimplePPT scales better with similar heuristics for trees with a higher number of nodes (up to 2000, Supplementary Fig. S1A), at the expense of the possibility of a less consistent tree output. To leverage the advantages of probabilistic mapping, scFates can also learn a principal graph on CellRank output by considering the combination of the absorption probabilities of each fate and calculated pseudotime as a manifold reduction, from which can be applied SimplePPT. The latter is relevant in cases where CellRank is more successful in capturing terminal fates than principal graph methods. The resulting trajectory, composed of connected principal points, can be projected onto any low-dimensional space, such as UMAP or ForceAtlas2. To orient a reconstructed trajectory, the user can choose one or two roots manually or according to the value of a feature, such as the level of expression of a known marker. Pseudotime positions of individual cells are then determined by projecting cells according to their position relative to the principal points (Fig. 1A). To account for uncertainty of a cell’s position along the trajectory, scFates leverages the soft assignment approach of SimplePPT. For that, each cell is assigned a probability to each principal point, which scFates can use to generate several pseudotime mappings to account for variability. In addition, scFates provides convenient functions for selecting specific portions of the tree, by selecting starting and endpoints, or by using pseudotime.

**Fig. 1.**
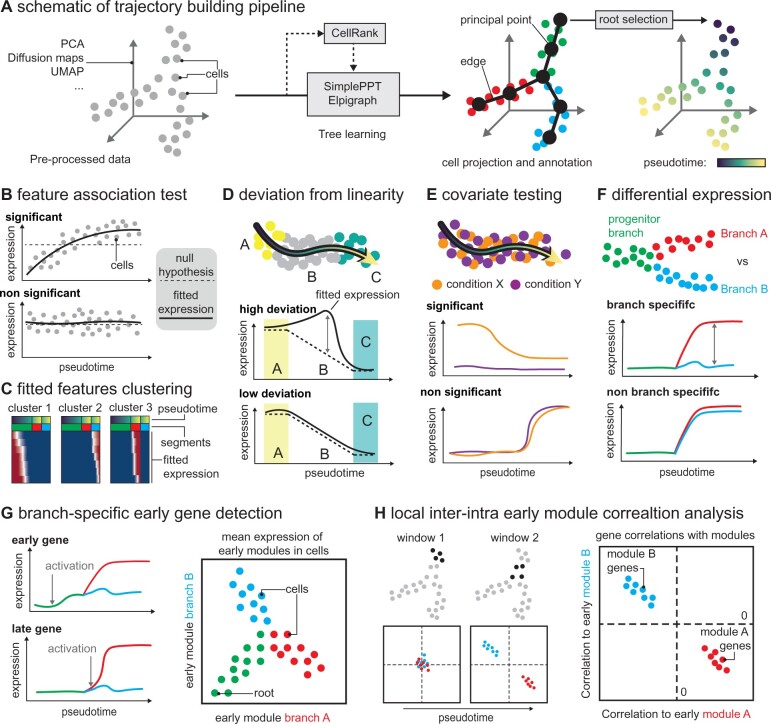
Schematic overview of scFates functionalities: (**A**) Dataset is first pre-processed via commonly used tools such as scanpy, to generate a reduced space where the tree will be learned using either ElPiGraph, SimplePPT or a combination of CellRank absorption probabilities and SimplePPT. After root selection, pseudotime and segment assignment is then calculated for each cell. (**B**) Features are tested for association with that pseudotime ordering using GAM models. (**C**) Fitted features can be clustered and visualized in different ways. (**D**) Transitions can be tested for deviation from linearity, to assess whether the bridge is likely to be an artifact and reveal transient features. (**E**) When two or more conditions are present, difference of amplitude or trends between conditions can be tested. (**F**) Branch-specific features (transcript count, ATAC-peak,…) are detected using GAM models. (**G**) A branch-specific feature (transcript count, ATAC-peak,…) is considered early if it displays activation dynamics prior to fork (left), early gene module mean expression can be used to detect co-activation of module prior to fork (right). (**H**) Early biasing analysis is done by calculating local gene-to-gene correlation of the detected early gene modules, before and around the fork. A separation of the two gene modules before the fork indicates repulsion of gene modules

We chose generalized additive models (GAM) as a framework for feature association testing, branch-DE and covariate analyses. Such models are well suited to capture non-linear gene trends in single-cell data and can provide amplitude measurement for prioritizing markers. Feature (e.g. gene expression and ATAC peaks) association testing is performed in a branch-specific manner for trees (Fig. 1B). We have performed benchmarks of the GAM model method for feature testing comparing to the alternative approaches implemented in STREAM and Monocle3. We found that while Monocle3’s Moran’s I does not yield amplitude measurements, that test can run faster as compared to GAM and agrees in terms of the detected features (Supplementary Fig. S1E and F). We have included wrappers for Monocle3’s approach to provide a choice to a user for feature testing. Associated features can be fitted and smoothed over pseudotime using the same GAM model. Smoothed features can be visualized and clustered using a distance metric of choice (Fig. 1C). In some cases, doublets can create transitions or bridges between two stationary populations. To detect such a situation, scFates incorporates a test based on deviations from linearity. Such a test consists of checking whether the expression dynamics observed in the bridge can be explained by a linear mixture of the flanking populations (Fig. 1D, Supplementary Fig. S2), such a test can be used to assure that the bridge is not an artifact, and uncover molecular transient features associated with these transitions (Kameneva *et al.*, 2021). Additionally, a function specifically designed for covariate testing is proposed to test for differential gene expression between conditions on the same trajectory path. This test includes an amplitude difference part, as well as trend differences (Fig. 1E, Supplementary Fig. S3).

Finally, scFates implements a set of functions for detailed analysis of bifurcations. First, early and late gene modules separating within each branch are detected by testing for differential expression between branches, and determining the timing of their deviation relative to the bifurcation (Fig. 1F and G, Supplementary Fig. S5). scFates then priorities features of early modules that contribute to cell fate biasing prior to the bifurcation point through backtracking of their correlations in the progenitor branch (Fig. 1H, Supplementary Fig. S5).

In conclusion, scFates is a fast and versatile tool for in-depth tree learning, pseudotime analysis and characterization of bifurcation dynamics from single-cell data. It can be widely applied in developmental biology, disease trajectory or perturbation analysis. Moreover, scFates can work with any type of highly dimensional data, allowing it to use with modalities other than gene expression, such as single-neuron activity over time.

## Supplementary Material

btac746_Supplementary_Data
